# Prediction of Protein Concentration in Pea (*Pisum sativum* L.) Using Near-Infrared Spectroscopy (NIRS) Systems

**DOI:** 10.3390/foods11223701

**Published:** 2022-11-18

**Authors:** Sintayehu D. Daba, David Honigs, Rebecca J. McGee, Alecia M. Kiszonas

**Affiliations:** 1 USDA-ARS Western Wheat Quality Laboratory, E-202 Food Quality Building, Washington State University, Pullman, WA 99164, USA; 2 PerkinElmer Inc., Waltham, MA 02451, USA; 3 USDA-ARS Grain Legume Research Unit, Washington State University, Pullman, WA 99164, USA

**Keywords:** protein prediction, dumas method, DA7250 system, FT9700 systems, PLSR, NIRS, spectral pre-treatment, calibration size

## Abstract

Breeding for increased protein concentration is a priority in field peas. Having a quick, accurate, and non-destructive protein quantification method is critical for screening breeding materials, which the near-infrared spectroscopy (NIRS) system can provide. Partial least square regression (PLSR) models to predict protein concentration were developed and compared for DA7250 and FT9700 NIRS systems. The reference protein data were accurate and exhibited a wider range of variation (15.3–29.8%). Spectral pre-treatments had no clear advantage over analyses based on raw spectral data. Due to the large number of samples used in this study, prediction accuracies remained similar across calibration sizes. The final PLSR models for the DA7250 and FT9700 systems required 10 and 13 latent variables, respectively, and performed well and were comparable (R^2^ = 0.72, RMSE = 1.22, and bias = 0.003 for DA7250; R^2^ = 0.79, RMSE = 1.23, and bias = 0.055 for FT9700). Considering three groupings for protein concentration (Low: <20%, Medium: ≥20%, but ≤25%, and High: >25%), none of the entries changed from low to high or vice versa between the observed and predicted values for the DA7250 system. Only a single entry moved from a low category in the observed data to a high category in the predicted data for the FT9700 system in the calibration set. Although the FT9700 system outperformed the DA7250 system by a small margin, both systems had the potential to predict protein concentration in pea seeds for breeding purposes. Wavelengths between 950 nm and 1650 nm accounted for most of the variation in pea protein concentration.

## 1. Introduction

Pulse crops are important for human consumption due to their high protein concentration and essential amino acids, as well as their role in preventing or controlling diseases such as cardiovascular diseases, type II diabetes, and cancer [1]. Pea is consumed primarily as a source of protein in a variety of forms, including as a vegetable, split pea, pea flour, protein isolates, and texturized protein products. Depending on the cultivar grown, environmental conditions, and testing method, protein concentration in peas can vary from 13% to 38% [2]. In pea breeding, high protein concentration is an important selection criterion. As a pea breeding program evaluates hundreds of lines, having a quick and precise method to measure protein concentration is required.

Near-infrared spectroscopy (NIRS) technology is useful for rapid, accurate, and non-destructive quantification of seed compositions [3], and it allows for the simultaneous determination of several parameters from a single measurement of spectral data [4] NIRS has been used in a variety of fields, such as the petrochemical, pharmaceutical, environmental, clinical, agricultural, food, and biomedical sectors [5]. It has been used to predict various seed components, including protein, oil, and amylose concentrations [6,7,8,9]. The NIRS technique uses spectral ranges between 780 nm and 2500 nm (12,500 cm^−1^ to 4000 cm^−1^) to offer information on the vibrational characteristics (stretching or bending) of molecular bonds such as O-H, C-H, C-O, and N-H [10]. These molecular bonds, which could be parts of proteins, exhibit absorption peaks at specific spectral wavelengths [11].

Before employing NIRS technology for routine seed composition measurements, it is important to build a good NIRS calibration. Calibration entails generating both reference and spectral data and utilizing chemometrics to develop predictive models based on the reference and spectral data [10]. When building a NIRS calibration, the precision and accuracy of both reference and spectral data are critical. Partial least squares regression (PLSR) is one of the most widely used chemometrics techniques for NIRS calibration [9,12,13]. When the number of predictors (for example, NIRS wavelengths) is larger than observations (sample size), ordinary multiple regression cannot be used. Two techniques could be used to deal with this problem, i.e., variable selection (spectral wavelength selection) and data projection [5]. Partial least squares regression is a data projection approach that makes use of uncorrelated and linear latent variables, which are also referred to as components or factors, created from combinations of the original spectral data [3,5,14]. Spectral pre-treatment or pre-processing may be required to take care of scattering effects due to differences in particle size among the samples [10,11,14,15]. Multiplicative scattering correction (MSC), standard normal variate (SNV) transformation, and Savitzky–Golay smoothing are some of the current spectral data pre-processing methods available [10,14,15,16].

As NIRS calibrations are intended to be applied to samples that are not used in the calibration process, it is vital to carefully validate the model prior to practical application. Validation approaches such as internal and external validations have been applied to develop best-performing models [12]. Particularly, the use of an independent sample set is crucial for efficient validation and to pave the way for the application of the NIRS calibrations in routine evaluations [14]. The predictive performance of the NIRS models is evaluated using a variety of statistics, including root mean square error prediction (RMSEP), coefficient of determination (R^2^), bias, and model efficiency (ME) estimation [3,9,12,17,18,19]. Optimum NIRS calibration models are selected when higher R^2^ and ME, but lower RMSEP and bias are achieved [3,12,20]. Optimizing the calibration parameters such as the size of the training (calibration) set and the number of latent variables for the PLSR model is critical. A comparison of the performance of NIRS calibrations from the different NIRS systems (DA7250 and FT9700) could be crucial to determine which system to use. Hence, the objective of this paper is to evaluate NIRS models developed for protein concentration in peas. Meanwhile, we investigated the impact of the training (calibration) population size and spectral pre-treatment on model predictive performance.

## 2. Materials and Methods

### 2.1. Pea Samples and Preparation of Whole Pea Flour

A total of 840 pea samples were obtained from different yield trials grown in 2020 and 2021 conducted in three U.S. states (North Dakota, Idaho, and Washington). The samples comprised diverse materials in seed size, colors (green vs. yellow), and growing habits (spring vs. winter). Details of the samples are presented in Table 1. Whole pea flour (particle size of 0.8 mm), prepared using a 3100 Perten Lab Mill (Perten Instruments, Hägersten, Sweden), was used to perform all the reference measurements.

### 2.2. Reference Measurements

An FP-528 dumas nitrogen analyzer (LECO Corporation, St. Joseph, MI, USA) was used to quantify protein concentration (N × 6.25) in duplicates using 0.10–0.19 g samples of whole pea flour. As a quality control of the FT-528 dumas nitrogen analyzer, three samples of Ethylenediaminetetraacetic acid (EDTA) were routinely quantified for protein concentration. We retrieved 36 protein concentration measurements for EDTA taken over the course of 12 batches of analyses. Protein concentrations of two cultivars (cv ‘Banner’ and cv ‘DS Admiral’) grown at Fairfield, WA in 2020 were also measured multiple times (n = 12) to check the repeatability of the FP-528 dumas nitrogen analyzer. A TGA701 thermogravimetric oven (LECO Corporation, St. Joseph, MI, USA) was used to measure moisture content and flour ash concentration in a 1.4–1.7 g sample of whole pea flour.

### 2.3. Scanning in DA7250 and FT9700 Machine for Spectral Data

All 840 samples were scanned using DA7250 and FT9700 NIRS machines (PerkinElmer Inc., Shelton, CT, USA) to generate spectral data. Scanning was performed on whole seed samples using a small rotating tray. The FT9700 covers a large spectral, ranging from 14,296 cm^−1^ to 3856 cm^−1^ (equivalent to 699.5 nm to 2593.4 nm) recorded at an 8 cm^−1^ resolution and making a total of 1306 wavelengths. For DA7250, spectral data were taken every 5 nm for a total of 141 wavelengths spanning 950 nm to 1650 nm. Each sample was scanned 32 times and the average was recorded as spectral data for each wavelength.

### 2.4. Data Analysis

The data analysis was separately performed for reference and spectral data as well as combining the two data types to develop predictive models. The least-square means (LSMEANS) for each sample were calculated using SAS 9.4 (SAS Institute Inc., Cary, NC, USA), which were then used for descriptive statistics, to create a distribution graph, and finally in the NIRS calibrations. Principal component analysis was performed using spectral data from DA7250 and FT9700 NIRS systems separately. The first two principal components were used to create a two-dimensional scatter plot and samples were clustered according to growing seasons (2020 and 2021) and protein categories (Low: <20%, Medium: ≥20% but ≤25%, and High: >25%).

The “pls” package [21] implemented in RStudio (RStudio team, 2020) was used to build NIRS calibration models. The pea samples (n = 840) were divided into calibration and validation subsets, with two methods used to achieve the classification. In the first method, we used the samples from one year as a calibration set and the other year as a validation set. In the second method, we utilized the Kennard-Stone sampling method [22] as implemented in the “prospectr” package [23] to divide the samples into the calibration and validation sets. The method starts with finding two samples farthest apart from each other and these two samples are then removed from the original data and put into the calibration set. This process is repeated until the desired calibration population size is reached. Five proportions calibration sets (50%, 60%, 70%, 80%, and 90%) of the original data were considered in this method. The root-mean-squared error (RMSE), coefficient of determination (R^2^), and bias values were used to determine the optimum size of the calibration set.

Plots of root mean squared error of prediction (RMSEP) against the number of components were used to determine the optimum number of latent variables to use in the models. The optimum number of latent variables was selected when the RMSEP reaches the lowest value or the change in RMSEP becomes smaller. Seven spectral data pre-treatment approaches were compared with raw-spectral-based analyses. Savitzky–Golay (SG), standard normal variate (SNV), SNV-detrend (SNV-D), and multiplicative scattering correction (MSC) as well as the combination of SG with the later three (SG + SNV, SG + SNV-D, and SG + MSC) were considered for spectral pre-treatment [10,14,15,16]. For FT9700, the selection of spectral data (950 nm to 1650 nm) was also considered to match the spectral range for the DA7250. After visually checking the pattern of spectral data, any sample with a noisy pattern was excluded. Overall, 32 different models were developed and compared, i.e., 14 for DA7250 and 18 for FT9700 NIRS machines. The four additional models for FT9700 were based on a truncated spectral range that corresponded to the spectral range of DA7250. After a thorough comparison of the models, the final two models (one for DA7250 and one for FT9700) were built with the optimal number of latent variables, the optimum calibration population size, appropriate spectral pre-treatment (if required), and excluding samples with noisy spectral patterns (if any). Parameters such as RMSE, R^2^, bias, and model efficiency (ME) were used to evaluate the models. The model efficiency (ME) was calculated using the formula described by Chiozza et al. [19] to evaluate the predictive ability of the models.

$$\mathrm{Model}\;\mathrm{efficiency}\;(\mathrm{ME})=1-\frac{\sum_{i=1}^{N}{\left(O_{i}-P_{i}\right)}^{2}}{\sum_{i=1}^{N}{\left(O_{i}-µ\right)}^{2}}$$

where O_i_ is the observed values, P_i_ is the predicted values, N is the number of samples, and µ is the mean of observed values.

## 3. Results and Discussion

### 3.1. Accuracy of the Reference Method

**Figure 1:**
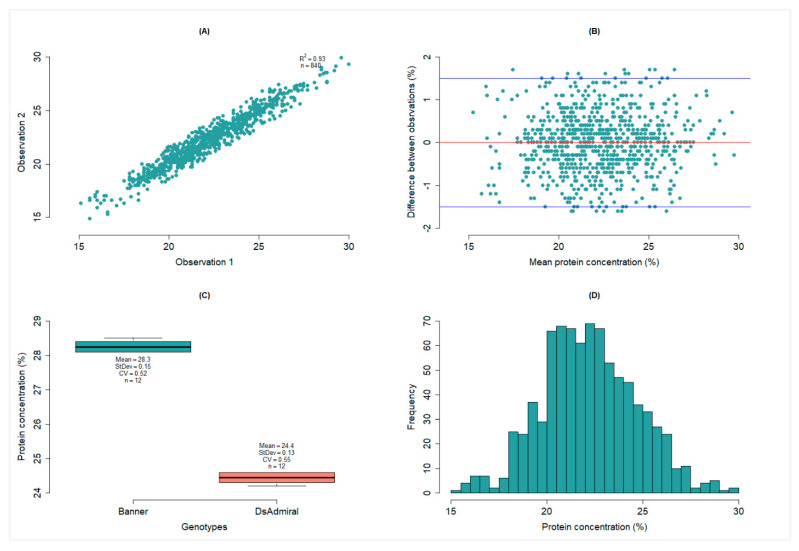
Diagnostic plots of reference data: (**A**) scatter plots of protein concentration observation 1 against observation 2, (**B**) plot of the differences between pair of observations against mean of each entry, (**C**) boxplots of multiple measurements (n = 12) of protein concentration for two pea genotypes (Banner and Ds Admiral) sampled from yield trial conducted at Fairfield, WA in 2020, and (**D**) distribution of protein concentration for the entries.

It is essential to collect the reference data using the best practices available. Hence, the first step in developing a robust NIRS calibration must be verifying the validity of the reference method, because poor reference methods lead to poor prediction accuracy [11,14]. The FP-528 dumas nitrogen analyzer (LECO Corporation, St. Joseph, MI, USA) was used to quantify the reference protein concentrations. We used different criteria to evaluate the reproducibility of the FP-528 dumas nitrogen analyzer in quantifying protein concentration. Figure 1A shows a two-dimensional plot of pairs of observations for all entries (n = 840), with the result indicating that the pairs of observations were well-matched (R^2^ = 0.93). The differences between the pairs of observations were typically within ±1.5% of each other (Figure 1B), with only 18 of the 840 entries falling outside of this range.

Using EDTA samples (three replications in a single batch of analysis), we checked the proper operation of the FP-528 dumas nitrogen analyzer before processing additional samples for protein concentration. The minimum, mean, and maximum protein concentrations for the 36 EDTA samples analyzed in 12 batches with the FP-528 dumas nitrogen analyzer were 54.2%, 54.6%, and 54.8%, respectively (Table 2), with a coefficient of variability (CV) of 0.17% (data not shown). CV can be used to assess the reproducibility of a procedure, and it can be interpreted as exceptional (0.5–1.0%), excellent (1.1–2.0%), very good (2.1–3.0%), good (3.1–4.0%), fair (4.1–5.0%), and needs investigation (≥5.1%).

In the third case, we used multiple measurements (n = 12) of two pea samples (cv ‘Banner’ and cv ‘DS Admiral’ grown at Fairfield, WA in 2020) to assess the repeatability of the FP-528 dumas nitrogen analyzer. Banner had a mean protein concentration of 28.3% with a CV of 0.50%, whereas DS Admiral had a mean protein concentration of 24.4% with a CV of 0.55% (Figure 1C). Overall, the results from all three cases demonstrated that the FP-528 dumas nitrogen analyzer had good repeatability in measuring protein concentration, and the reference data are appropriate for NIRS calibration.

### 3.2. Variation among Pea Samples for the Reference Data

The reference protein concentration data showed a wide range of variation (Table 2 and Figure 1D), which is required for NIRS calibration. The mean protein concentration for the reference data was 22.3%, with a low of 15.3% and a high of 29.8%, which was within the range reported previously [2]. The CV calculated for protein concentration based on means of entries was 11.2% (data not shown), implying that there was a wider level of variability among the entries. Moisture and ash concentrations of the flour samples were also collected. The moisture concentration ranged from 7.4% to 9.4% with a mean of 8.1%, whereas the ash concentration ranged from 1.8% to 3.0% with a mean of 2.5% (Table 2). Narrow variability in the reference data negatively impacts the accuracy of NIRS prediction [14]. As both moisture content and ash concentration had a small range of variation, they were excluded from the NIRS calibration.

**Figure 2:**
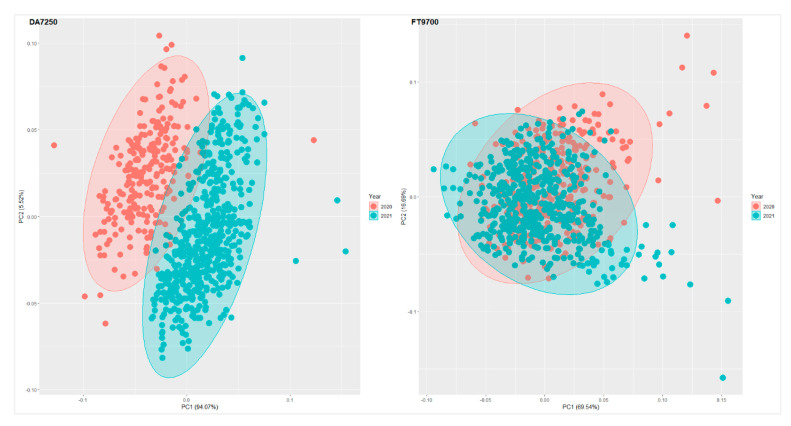
Clustering of pea samples based on the first and second principal components generated using DA7250 and FT9700 spectral data.

The plots in Figures S1 and S2 can be used to compare visually the effect of the different spectral pre-treatments with raw spectral data. In the case of FT9700 spectral data, one entry (P21_02_0254) displayed a distinct spectral pattern across the entire range of spectral bands (Figure S2). Excluding this sample from the calibration process may be beneficial. The first two components from spectral-data-based principal component analyses accounted for 99.6% and 86.2% of the variation for DA7250 and FT9700 systems, respectively (Figure 2). The samples were mainly grouped by growing season, specifically for the DA7250 spectral data. The protein concentration data also revealed year-to-year variations. When the locations included in both years were considered, the mean and variability of protein concentrations were found to be higher in 2021 as compared with 2020 (Table 3). In 2020, Fairfield, WA had the highest mean protein concentration (23.3%). Colton, WA had the highest mean protein concentration (25.7%) in 2021, followed by Fairfield, WA (23.3%).

### 3.3. Comparing Models Developed Year-Wise and by Kennard-Stone Sampling

For the DA7250 system, a NIRS model developed using samples from 2020 as the calibration set resulted in R^2^ values of 0.86 and 0.71 in the calibration and validation sets with root mean square error (RMSE) of 0.73 and 1.80, respectively (Table 4). When the samples from 2021 were used as a calibration set in the DA7250 system, the R^2^ increased to 0.75 and RMSE decreased to 1.11 in the validation set. According to Williams et al. [11], R^2^ values greater than 0.66 are acceptable for screening whereas values less than 0.50 are not suitable for NIRS calibration. Both year-wise models for DA7250 could be sufficient for screening breeding materials for protein concentration. However, with the FT9700 system, the calibration for protein concentration developed based on samples from one year to predict the other year was shown to be inadequate, resulting in large bias values (Table 4). The R^2^ values for a model built using 2020 as the calibration set in the FT9700 system were 0.89 in the calibration set and 0.65 in the validation set, with RMSE values of 0.64 and 6.0, respectively. In general, this model led to a larger bias in the validation set (5.9). The use of 2021 samples as a calibration set for the FT9700 system increased the R^2^ value in the validation set to 0.78. Despite this improvement in R^2^, the bias value of this model was found to be high (−2.25).

We evaluated five PLSR models with varying calibration to validation ratios (1:1, 3:2, 7:3, 4:1, and 9:1), which correspond to 50%, 60%, 70%, 80%, and 90% of the total samples in the calibration set, respectively (Table 4). For all five calibration population sizes, the R^2^ values for the DA7250 system were comparable, ranging from 0.81 to 0.85 in the calibration set and from 0.71 to 0.76 in the validation set. Similarly, comparable R^2^ values were observed for calibration (0.81 to 0.86) and validation (0.69 to 0.72) sets among the five calibration population sizes in the FT9700 system. For both the DA7250 and FT9700 systems, RMSE and bias values also demonstrated comparable prediction accuracies across the five calibration population sizes. The lack of significant differences in prediction accuracies across calibration sizes could be attributed to the larger original samples (n = 840) used in this study. The final prediction models may be developed using any of these calibration sizes, but we chose the 70-30 ratio because it generated the lowest bias in the validation set in both the DA7250 (bias = 0.003) and FT9700 (bias = 0.005) systems (Table 4). Overall, the models developed using Kennard-Stone sampling were better than the models developed year-wise, as evident from bias values (Table 4).

### 3.4. Comparison of Spectral Pre-Treatment with Raw-Spectral Models

We compared the results of pre-treated spectral data with raw-spectral-based analyses. Spectral pre-treatment is usually suggested in NIRS chemometrics to account for scattering effects caused by differences in particle size of the samples [10,11,14,15]. This study considered individual approaches such as Savitzky–Golay smoothing (SG), standard normal variate (SNV), SNV detrend (SNV-D), and multiplicative scattering correction (MSC), as well as combinations of SG with SNV, SNV-D, and MSC. Models are evaluated based on how well they performed, particularly in the validation set.

Both pre-treatment and raw-spectral analyses were performed using a 70% calibration population size. The pre-treatment methods in general provided higher R^2^ (0.76–0.80) in the validation set in the DA7250 system, with the highest R^2^ value from the model developed using the combination of standard normal variate detrending and Savitzky–Golay smoothening (Table 4). The R^2^ in the validation set for raw-spectral-based analysis for the DA7250 system was 0.73. Similarly, the pre-treatment had higher R^2^ compared with raw spectral-based analysis in the FT9700 system (0.74–0.81 vs. 0.69). In the raw-spectral analyses, the R^2^ was increased to 0.79 by simply excluding P21_02_0254 (the entry with a distinct spectral pattern). Contrary to R^2^, the lowest bias values in the validation set were recorded for raw-spectral-based analyses. Despite the slight differences, neither NIRS system demonstrated a clear advantage of spectral pre-treatment over raw-spectral analyses. In such cases, raw-spectral analyses are preferable because some spectral pre-treatment procedures such as Savitzky–Golay smoothening trim the outer spectral bands. Thus, we built the final models using raw spectral data and 70% of the samples in the calibration set. In the case of the FT9700 system, we also excluded P21_02_0254.

### 3.5. Final Models and Principal Absorption Wavelengths

**Figure 3:**
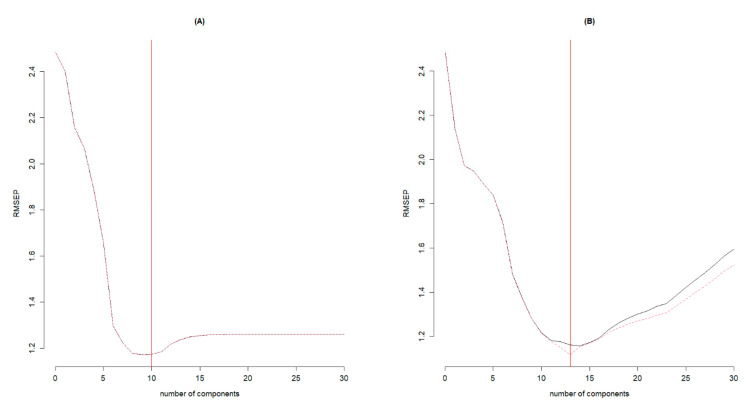
Plots of root mean squares error of prediction (RMSEP) against the number of components: (**A**) for partial least square regression (PLSR) using DA7250 spectral data, and (**B**) for PLSR using FT9700 spectral data. The red vertical line indicates the number of components used in the final models.

**Figure 4:**
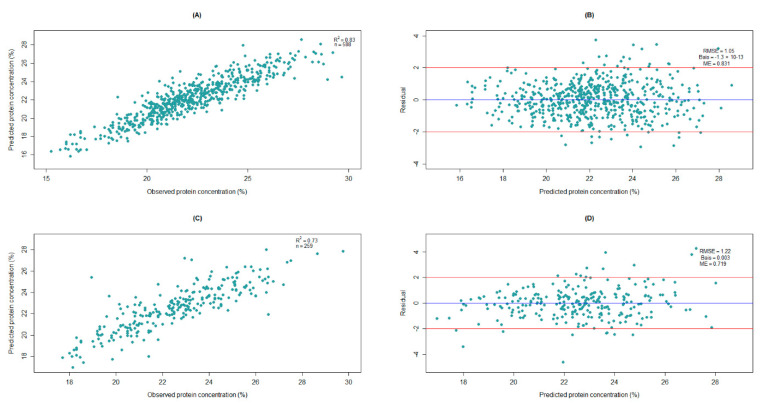
Different plots for partial least square regression (PLSR) using DA7250 spectral data: (**A**) scatter plot of predicted values against observed values for the calibration set, (**B**) plot of residual values against predicted values for the calibration set, (**C**) scatter plot of predicted values against observed values for the validation set, and (**D**) plot of residual values against predicted values for the validation set.

The final model for the DA7250 system required 10 latent variables (Figure 3A) and it accounted for approximately 83% of the variation in the calibration set (Figure 4A) and 73% of the variation in the validation set (Figure 4C). Residual values, which are the difference between observed and predicted values, were mainly within ±2.0% in both the calibration and validation sets (Figure 4B,D). Only 5.9% and 6.7% of the entries were found to have residuals beyond ±2.0% in the calibration and validation sets, respectively. The RMSE values were 1.05 for the calibration set and 1.22 for the validation set (Figure 4B,D), which represented 4.7% and 5.5% of the mean protein concentration (22.3%). Model efficiency (ME) values closer to 1.0 indicate the better predictive ability of the model [19], which were 0.83 and 0.72 for the calibration and the validation sets, respectively, in our study (Figure 4B,D).

The utility of NIRS calibration for routine varietal screening can also be determined by comparing the change in category for entries between observed and predicted values. For this purpose, we divided the protein concentration data into three categories: low protein (<20%), medium protein (≥20% but ≤25%), and high protein (>25%). At the boundaries of the three categories, a category switch between observed and predicted values is logically expected (that is, entries can switch from/to low or high categories from/to medium category). However, shifting from low to high or vice versa in many of the entries may indicate a serious flaw in the model. In both the calibration and validation sets, none of the entries jumped from low to high or vice versa between the observed and predicted values in the DA7250 system (Table 5).

**Figure 5:**
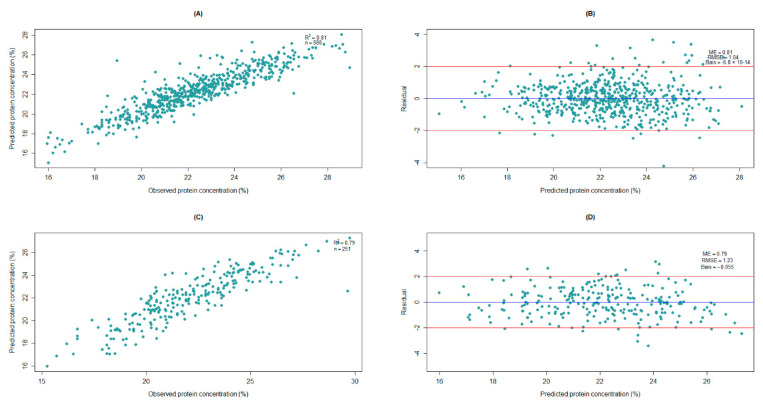
Different plots for partial least square regression (PLSR) using FT9700 spectral data: (**A**) scatter plot of predicted values against observed values for the calibration set, (**B**) plot of residual values against predicted values for the calibration set, (**C**) scatter plot of predicted values set against observed values for the validation, and (**D**) plot of residual values against predicted values for the validation set.

For the FT9700 system, the final model with 13 latent variables was found to be optimum (Figure 3B) and this model accounted for 81% of the variation in the calibration set (Figure 5A) and 79% of the variation in the validation dataset (Figure 5C). The residual values were within ±2.0% for most of the entries (Figure 5B,D), with only 4.9% and 8.4% of the entries out of this range in the calibration and validation sets, respectively (data not shown). The RMSE values were 1.04% in the calibration set and 1.23% in the validation set, representing 4.7% and 5.5% of the mean observed protein concentration (22.3%), respectively. The ME estimates were 0.81 and 0.79 for the calibration and validation sets, respectively. All model performance parameters suggested that the FT9700 system slightly outperformed the DA7250 system in predicting protein concentration in peas. When spectral data from 950 nm to 1650 nm in the FT9700 system (which is equivalent to the spectral range of DA7250) were considered, its predictive performance was comparable to the model with the entire range of wavelengths but excluding P21_02_0254 (Table 3). This could imply that spectral wavelengths ranging from 950 nm to 1650 nm played a larger role in the variation of protein concentration in peas. Evaluation of the shift in the category between observed and predicted values for the FT9700 system revealed that only one entry moved from a low in observed to a high in predicted for the calibration set (Table 5).

Near-infrared spectroscopy has been applied to predict protein concentration in a variety of pulse crops, including peas [24,25,26,27], peanuts [28], Faba beans [24], and chickpeas [24]. Williams et al. [24] calibrated the NIRS from the Neotec Grain Quality Analyzer (model 31) to protein concentration (N × 6.25) determined by the Kjeldahl test for various pulse crops. They reported R^2^ values ranging from 0.89 for dry peas to 0.96 for broad beans, Faba beans, and vetch. The RMSE estimates for the same study were ranging from 0.37 for vetch to 0.78 for the broad bean. Our pea protein prediction models were slightly less powerful than those reported by Williams et al. (1978) [24], which could be attributed to the fact that we used whole seed samples whereas they used ground samples. NIRS calibrations based on ground samples usually outperformed calibrations based on intact seed samples [27,28,29]. The use of intact seed samples, on the other hand, is advantageous for breeding as it is non-destructive and many lines must be evaluated in a short period of time. In this respect, our NIRS calibrations for both systems could be useful for screening pea breeding lines.

**Figure 6:**
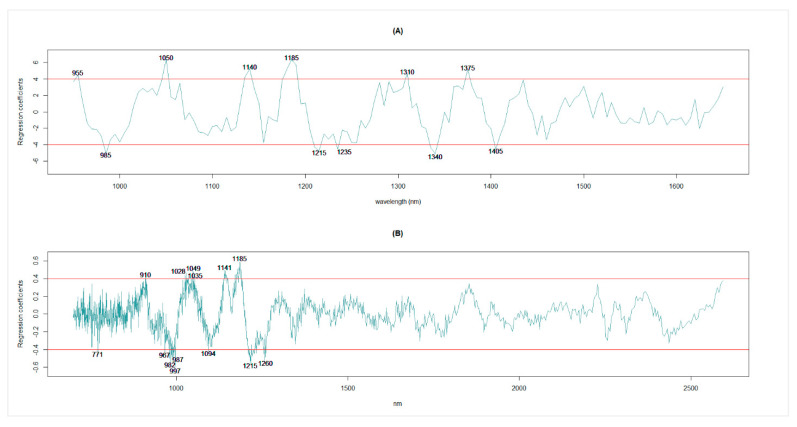
Plots of regression coefficient for the PLSR models with optimum number of latent variables (10 for DA7250 and 13 for FT9700) against spectral wavelengths (950 nm to 1650 nm for DA7250 and 699.5 nm to 2593.4 nm for FT9700). The models were: (**A**) PLSR using DA7250 spectral data and (**B**) PLSR using FT9700 spectral data. The red horizontal line indicates threshold to declare high regression.

The different functional groups (C-H, O-H, N-H, C-N, C-O, S-H, and P-H) of biological molecules show absorption at specific wavelengths [11], which could be visualized from the regression coefficient plots. Spectral wavelength regions with high regression coefficients could be useful in this respect. The regression coefficients for final models for the DA7250 system using 10 latent variables ranged from −5.11 to 6.43 (data not shown), with 11 spectral wavelength regions having regression coefficients ≥4.0 or ≤−4.0 (Figure 6A). Additional high regression regions were also detected at such wavelengths as 950 nm (3.70), 1000 nm (−3.64), 1155 nm (−3.78), 1175 nm (3.87), 1255 nm (−3.75), 1290 nm (3.69), and 1435 nm (3.90), which were not labeled in Figure 6A because the values were slightly short of the cur-point (≥4.0 or ≤−4.0).

In the case of FT9700, the regression coefficients using 13 latent variables ranged between −0.59 and 0.59 (data not shown), which were considerably lower than the regression coefficients of the model with DA7250 spectral data. This difference could partly be explained by a larger number of spectral wavelengths for FT9700 than for the DA7250 (1306 vs. 141). When only 950 nm to 1650 nm (535 wavelengths) are selected for the FT9700 system, the regression coefficients were between −0.94 and 0.89. Fourteen spectral wavelengths had regression coefficients ≥0.4 or ≤−0.4 in the final FT9700 model (Figure 6B). The FT9700 system also had additional regression coefficients slightly short of the cut-point (≥0.4 or ≤−0.4) at wavelengths 752 nm (−0.37), 1101 nm (−0.37), 1176 nm (0.39), and 2593 nm (0.38). Eight of them (987 nm, 997 nm, 1049 nm, 1141 nm, 1175 nm, 1185 nm, 1215 nm, and 1260 nm) were within ±5 nm of the regions identified in the DA7250 system as having high regression coefficients.

The larger regression coefficients were mainly centered between 950 nm and 1435 nm in the DA7250 system and between 752 nm and 1347 nm in the FT9700 system, which was in line with that reported by Hacisalihoglu et al. [8]. Hacisalihoglu et al. [8] reported high regression coefficients for protein concentration for peas at 910 nm, 996 nm, 1026 nm, 1189 nm, 1214 nm, and 1383 nm. Some of them coincided with spectral regions detected in this study (910 nm, 997 nm/1000 nm, 1028 nm, 1185 nm, 1215 nm, and 1375 nm). Williams et al. [11] summarized the spectral positions associated with common agricultural and food constituents (including protein, starch, and oil), some of which were also detected for protein in the current study (910 nm in DA7250, 982 nm in FT9700, 1140 nm in DA7250/1141 nm in FT9700, and 1185 nm in DA7250 and FT9700).

## 4. Conclusions

Near-infrared spectroscopy (NIRS) models have great potential to predict seed compositions quickly, reliably, and non-destructively. The models developed from two NIRS systems (DA7250 and FT9700) provided a good prediction of protein concentration in peas in the calibration and validation sets. Although the FT9700 systems slightly outperformed the DA7250, both can be used to predict protein concentration for screening pea breeding lines. Using flour samples generally provides a better prediction of protein [30]. However, avoiding the flour preparation process and using the intact seed saves time and allows for the quantification of large samples in time for a breeding program. In this regard, our models based on whole seed samples are beneficial to pea breeding programs.

Calibration population size, spectral wavelength selection, and spectral pre-treatments are usually recommended in the NIRS calibration process. Our study indicated that calibration population size had no effect on prediction performance, as our initial samples were large. As the particle size and moisture content difference contribute to the variations in near-infrared absorption, the pre-processing of spectral data is usually recommended. The pre-treatment methods we applied were not considerably superior in predictive performance over the raw-spectral data (spectral data without pre-treatment). Thus, spectral pre-treatment may not be necessary for this specific case to develop the most optimum NIRS models for protein concentration in peas. One of the differences between the DA7250 and FT9700 systems was the existence of wavelengths below 950 nm and above 1650 nm in the FT9700. The results from this study indicated that the extra wavelengths in the FT9700 contributed to a smaller extent in predicting pea protein concentration. Hang et al. [30] also reported a non-significant difference in the predictive ability of DA7250 and FT9700 for protein and amino acids in lentils.

Overall, when applying these calibration models in day-to-day operation, it is beneficial to include some check pea cultivars with protein concentration determined by a reference method at certain intervals in the NIRS measurement process. It is also vital to update the model regularly as more materials are included that have a wider range of protein concentrations than that covered in the current study.

## Figures and Tables

**Table 1:** Composition of pea samples utilized in the calibration of near-infrared spectroscopy to predict protein concentration.

Type	n	Growing States	Growth Habits	Year
GR pea samples	334	Washington	Spring	2020 & 2021
ID pea samples	42	Idaho	Winter	2020
ND pea samples	15	North Dakota	Spring	2020
Winter pea samples	231	Washington	Winter	2020 & 2021
YL samples peas	218	Washington	Spring	2020 & 2021

GR = Green, ID = Idaho, ND = North Dakota, YL = Yellow, n indicates the total number of samples in both years.

**Table 2:** Minimum, mean, and maximum protein concentration (%) for flour and EDTA, flour ash concentration (%), and moisture content (%).

Parameters	n	Minimum	Mean ± SD	Maximum
Flour protein (%)	840	15.3	22.3 ± 2.5	29.8
Flour ash (%)	840	1.8	2.5 ± 0.19	3.0
Flour moisture (%)	840	7.4	8.1 ± 0.32	9.4
EDTA protein (%)	36	54.2	54.6 ± 0.09	54.8

SD indicates standard deviation; n = number of entries for flour or samples for EDTA.

**Table 3:** Mean and variability in protein concentration by growing seasons and locations.

Locations	2020				2021			
	Mean	Minimum	Maximum	CV (%)	Mean	Minimum	Maximum	CV (%)
Colton	21.5	18.5	23.2	6.0	25.7	19.0	29.8	7.4
Fairfield	23.3	20.1	27.2	6.1	23.3	19.7	29.7	6.7
Garfield	18.6	16.0	22.5	9.9	19.6	15.3	24.8	10.7
Pullman	20.9	16.5	24.5	7.5	22.7	16.2	29.0	10.4
St. John	20.3	18.3	23.6	5.9	21.6	18.6	25.5	7.1
Williston	20.7	19.5	21.7	3.9				
Carrington	22.0	20.3	24.2	6.5				
Minot	20.4	18.3	22.1	6.6				
Moscow	20.3	16.0	23.3	8.3				
Craigmond	20.4	18.3	22.4	5.9				
Dayton	20.2	18.0	22.3	5.5				
Overall	21.0	16.0	27.2	9.3	22.9	15.3	29.8	10.8

**Table 4:** Model evaluation parameters (R2, RMSE, and bias) for predictive models developed based on different considerations in the DA7250 and FT9700 NIRS systems.

	Pre-Treatment/Spectral Selection	Size of Calibration/Validation Sets	LVs	Calibration Set			Validation Set		
				R^2^	RMSE	Bias	R^2^	RMSE	Bias
		Calibration/Validation set size							
DA7250	none	CAL50 (n = 420)–VAL50 (n = 420)	10	0.85	0.97	−1.80 × 10^−14^	0.74	1.25	0.080
	none	CAL60 (n = 504)–VAL40 (n = 336)	10	0.85	0.99	−1.20 × 10^−13^	0.71	1.30	−0.030
	none *	CAL70 (n = 588)–VAL30 (n = 252)	10	0.83	1.05	−1.30 × 10^−13^	0.73	1.22	0.003
	none	CAL80 (n = 672)–VAL20 (n = 168)	10	0.82	1.07	−1.60 × 10^−13^	0.74	1.18	−0.074
	none	CAL90 (n = 756)–VAL10 (n = 84)	10	0.81	1.09	−3.60 × 10^−14^	0.76	1.15	0.093
FT9700	none	CAL50 (n = 420)–VAL50 (n = 420)	13	0.86	0.95	−1.20 × 10^−13^	0.71	1.31	−0.050
	none	CAL60 (n = 504)–VAL40 (n = 336)	13	0.86	0.97	−1.10 × 10^−14^	0.69	1.33	−0.007
	none	CAL70 (n = 588)–VAL30 (n = 252)	13	0.84	1.05	−4.30 × 10^−13^	0.69	1.28	0.005
	none (Entry P21_02_0254 removed) *	CAL70 (n = 588)–VAL30 (n = 251)	13	0.81	1.04	−6.80 × 10^−14^	0.79	1.23	−0.055
	none	CAL80 (n = 672)–VAL20 (n = 168)	13	0.82	1.08	−3.50 × 10^−13^	0.72	1.21	−0.071
	none	CAL90 (n = 756)–VAL10 (n = 84)	13	0.81	1.08	−1.80 × 10^−14^	0.70	1.28	0.130
		Spectral pre-treatment							
DA7250	SG	CAL70 (n = 588)–VAL30 (n = 252)	10	0.79	1.15	−1.40 × 10^−13^	0.77	1.16	−0.188
	SNV	CAL70 (n = 588)–VAL30 (n = 252)	7	0.80	1.13	−1.60 × 10^−13^	0.77	1.16	−0.176
	SNV-D	CAL70 (n = 588)–VAL30 (n = 252)	6	0.80	1.14	−3.20 × 10^−14^	0.76	1.17	−0.166
	MSC	CAL70 (n = 588)–VAL30 (n = 252)	7	0.79	1.12	−1.40 × 10^−13^	0.77	1.16	−0.188
	SNV + SG	CAL70 (n = 588)–VAL30 (n = 252)	9	0.80	1.11	−1.20 × 10^−13^	0.76	1.21	−0.080
	SNV-D + SG	CAL70 (n = 588)–VAL30 (n = 252)	8	0.78	1.17	−5.00 × 10^−14^	0.80	1.10	−0.011
	MSC + SG	CAL70 (n = 588)–VAL30 (n = 252)	9	0.80	1.11	−1.10 × 10^−13^	0.76	1.21	−0.095
FT9700	SG	CAL70 (n = 588)–VAL30 (n = 252)	13	0.82	1.08	−6.80 × 10^−13^	0.78	1.14	−0.204
	SNV	CAL70 (n = 588)–VAL30 (n = 252)	14	0.85	0.97	3.20 × 10^−14^	0.74	1.23	−0.193
	SNV-D	CAL70 (n = 588)–VAL30 (n = 252)	12	0.84	1.02	−1.10 × 10^−14^	0.76	1.18	−0.243
	MSC	CAL70 (n = 588)–VAL30 (n = 252)	13	0.86	0.95	−1.40 × 10^−14^	0.76	1.18	−0.217
	SNV + SG	CAL70 (n = 588)–VAL30 (n = 252)	15	0.83	1.04	−2.10 × 10^−14^	0.75	1.23	0.087
	SNV-D + SG	CAL70 (n = 588)–VAL30 (n = 252)	12	0.82	1.05	−2.50 × 10^−14^	0.81	1.06	0.022
	MSC + SG	CAL70 (n = 588)–VAL30 (n = 252)	14	0.83	1.11	−1.10 × 10^−13^	0.76	1.21	−0.095
		Calibration/Validation sets year-wise							
DA7250	none	CAL2020 (n = 299)–VAL2021 (n = 541)	10	0.86	0.73	−1.30 × 10^−13^	0.71	1.80	−1.13
	none	CAL2021 (n = 541)–VAL2020 (n = 299)	10	0.78	1.17	−1.10 × 10^−13^	0.75	1.11	−0.54
FT9700	none	CAL2020 (n = 299)–VAL2021 (n = 541)	13	0.89	0.64	−2.80 × 10^−14^	0.65	6.00	5.90
	none	CAL2021 (n = 541)–VAL2020 (n = 299)	13	0.78	1.16	−1.80 × 10^−14^	0.78	2.43	−2.25
		Spectral wavelength selection							
FT9700	950 nm to 1650 nm	CAL70 (n = 588)–VAL30 (n = 252)	9	0.80	1.1	7.10 × 10^−14^	0.80	1.11	−0.122
	950 nm to 1650 nm	CAL2020 (n = 299)–VAL2021 (n = 541)	9	0.86	0.73	−1.80 × 10^−14^	0.68	2.60	2.14
	950 nm to 1650 nm	CAL2021 (n = 541)–VAL2020 (n = 299)	10	0.80	1.12	−4.30 × 10^−13^	0.77	0.96	0.026

none means no pre-treatment or spectral selection used, LVs = latent variables, R^2^ = coefficient of determination, RMSE = root-mean-squared error, SG = Savitzky–Golay pre-treatment used, SNV = Standard normal variate pre-treatment used, SNV-D = Standard normal variate detrend pre-treatment used, MSC = multiplicative scattering correction pre-treatment used, 950 nm to 1650 nm indicates spectral data within this range was selected and used in the analysis, CAL = calibration set, VAL = Validation set, numbers following CAL and VAL such as 10, 20, 30, 40, 50, 60, 70, 80, and 90 represented the percentage of samples used in the calibration or validation sets, and numbers such as 2020 and 2021 represented 2020 and 2021 growing season, respectively. The models highlighted in bold and marked with an asterisk (*) were used as final models for the two NIRS systems.

**Table 5:** Level of misclassification of observed protein concentration (High and Low groups) after predicted with the final models for both DA7250 and FT9700 systems.

Datasets	Observed	Predicted		
		HIGH	MEDIUM	LOW
Calibration DA7250_CAL70_VAL30	HIGH	48	30	0
	LOW	0	19	87
Validation DA7250_CAL70_VAL30	HIGH	21	20	0
	LOW	0	12	24
Calibration FT9700_CAL70_VAL30	HIGH	50	34	0
	LOW	1	21	69
Validation FT9700_CAL70_VAL30	HIGH	18	17	0
	LOW	0	12	39

Note: The grouping was created based on the protein concentration values, i.e., LOW comprised of samples with protein concentration less than 20, MEDIUM comprised on samples with protein concentration 20% to 25%, HIGH comprised of the samples with protein concentration greater than 25%. The number in the table indicates the number of samples in each category.

## Data Availability

Data is contained within the article or Supplementary Material.

## Supplementary Materials

The following are available online at https://www.mdpi.com/article/10.3390/foods11223701/s1. Figure S1. Spectral data graphs of the raw-spectral and the different pre-treatment approaches for the DA7250 system; Figure S2. Spectral data graphs of the raw-spectral and the different pre-treatment approaches for the FT9700 system.
