# Supplementary material for: Prediction of Protein Concentration in Pea (Pisum sativum L.) Using Near-Infrared Spectroscopy (NIRS) Systems
Source: Foods. 2022 Nov 18;11(22):3701. doi: 10.3390/foods11223701 (PMC9689555; doi:10.3390/foods11223701)
Supplement: Supplementary file 1 [file foods-11-03701-s001.zip › foods-2008328-supplementary.pdf]

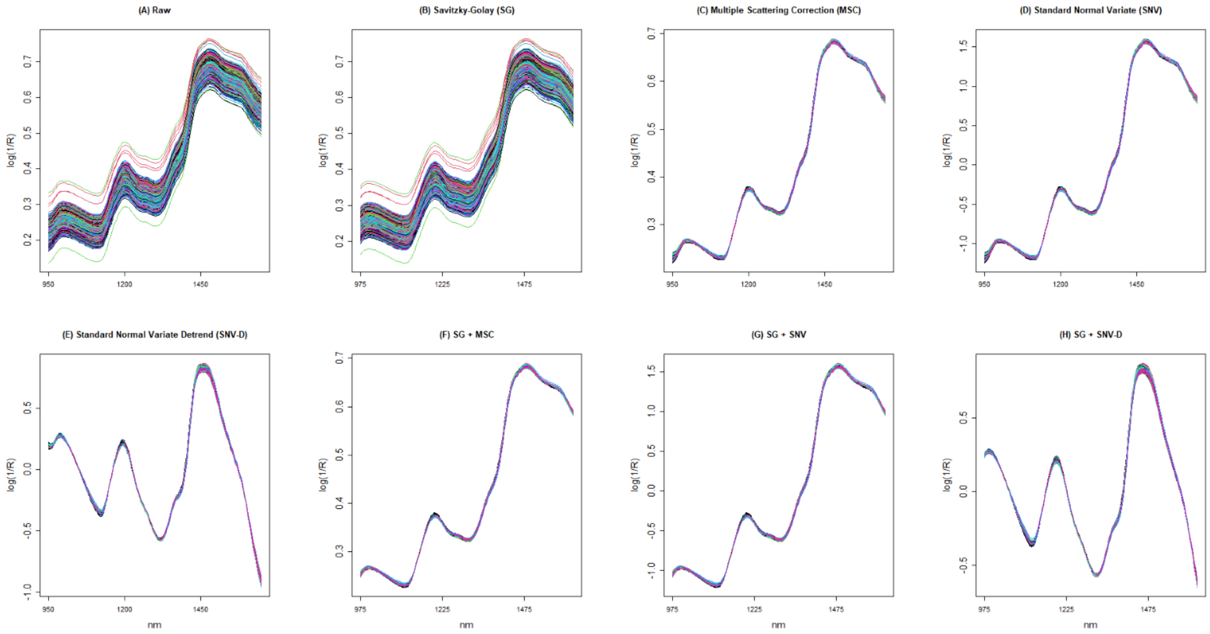

Figure S1. Spectral data graphs of the raw-spectral and the different pre-treatment approaches for the DA7250 system:(**A**) Raw, (**B**) Savitzky–Golay (SG), (**C**) Multiplicative Scattering Correction (MSC), (**D**) Standard Normal Variate (SNV), (**E**) Standard Normal Variate Detrend(SNV-D), (**F**) SG + MSC,:(**G**) SG + SNV,:(**H**) SG + SNV-D.

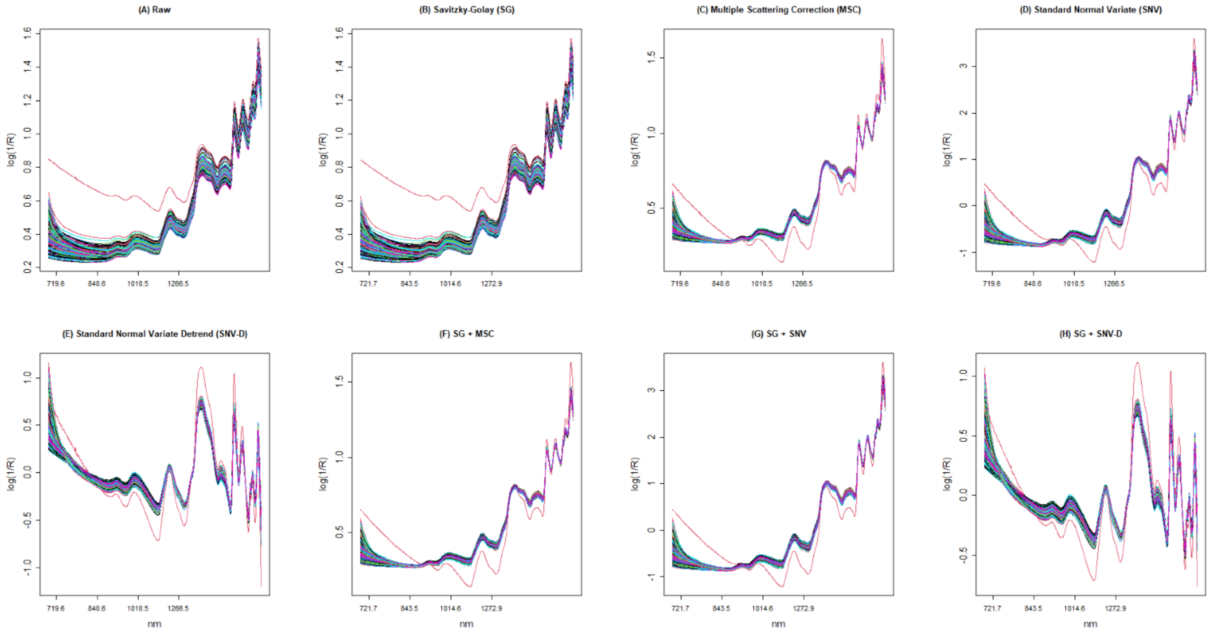

Figure S2. Spectral data graphs of the raw-spectral and the different pre-treatment approaches for the FT9700 system:(**A**) Raw, (**B**) Savitzky–Golay (SG), (**C**) Multiplicative Scattering Correction (MSC), (**D**) Standard Normal Variate (SNV), (**E**) Standard Normal Variate Detrend(SNV-D), (**F**) SG + MSC,:(**G**) SG + SNV,:(**H**) SG + SNV-D.
